# HPV integration as a triage tool for HPV-positive women in cervical cancer screening

**DOI:** 10.1016/j.tvr.2026.200351

**Published:** 2026-09-01

**Authors:** Xite Lin, Yue Wang, Nan Yu, Dingjie Wang, Liang Wang, Xiaoyuan Huang, Binhua Dong, Pengming Sun

**Affiliations:** a Laboratory of Gynecologic Oncology, Fujian Maternity and Child Health Hospital, Fujian Medical University, Fuzhou, Fujian, 350001, China; b College of Clinical Medicine for Obstetrics & Gynecology and Pediatrics, Fujian Medical University, Fuzhou, Fujian, 350001, China; c Fujian Key Laboratory of Women and Children's Critical Diseases Research, Fujian Maternity and Child Health Hospital, Fuzhou, Fujian, 350001, China; d Fujian Clinical Research Center for Gynecological Oncology, Fujian Maternity and Child Health Hospital, Fuzhou, Fujian, 350001, China; e Department of Obstetrics and Gynecology, Tongji Hospital, Tongji Medical College, Huazhong University of Science and Technology, 1095, Jiefang Road, Wuhan, Hubei, 430030, China; f Department of Gynecology, Fujian Maternity and Child Health Hospital, Affiliated Hospital of Fujian Medical University, Fuzhou, Fujian, 350001, China; g Department of Obstetrics and Gynecology, National Clinical Research Center for Obstetrics and Gynecology, Tongji Hospital, Tongji Medical College, Huazhong University of Science and Technology, Wuhan, China

**Keywords:** Human papillomavirus (HPV), HPV integration, Cervical cancer screening, Triage, Precision medicine

## Abstract

Effective triage of HPV-positive women remains challenging, as conventional cytology often yields excessive colposcopy referrals due to limited specificity. We evaluated HPV integration status-a key molecular marker associated with cervical carcinogenesis-as a novel triage biomarker within the Fujian-Hubei Screening Cohort (N = 31,628). Among 949 prospectively enrolled HPV-positive women with concurrent HPV integration testing (HIVID), genotyping, and cytology, HPV integration emerged as the strongest independent predictor of CIN3+ (aOR = 68.1). As a standalone triage tool, HPV integration detection significantly outperformed cytology, demonstrating superior specificity (97.0% vs. 64.3%; p < 0.001) and PPV (57.1% vs. 10.4%; p < 0.001) while maintaining comparable sensitivity (66.7% vs. 68.5%). Critically, our combined strategy integrating HPV16/18 genotyping with integration detection achieved optimized performance: it enhanced specificity by 25.2% (69.5% vs. 44.3%) and yielded a higher PPV (14.7% vs. 9.1%) compared to standard cytology + genotyping, with no significant sensitivity loss (87.0% vs. 92.6%; p = 0.526). This approach identified a large low-risk subgroup (NILM/integration-negative; 59.2% of cohort) with minimal CIN3+ risk (0.7%), enabling an 82% reduction in colposcopy referrals versus cytology triage (6.6% vs. 37.6%; p < 0.001). These results position HPV integration as a robust biomarker for precision risk stratification. The combined use of HPV genotyping and integration detection offers an efficient, clinically actionable strategy to minimize unnecessary procedures while maintaining high-grade lesion detection.

## 1 Introduction

Cervical cancer (CC) remains a major global public health challenge, accounting for approximately 7% of all female cancers, with an estimated 660,000 new cases and over 340,000 deaths annually [1]. The burden is disproportionately high in low-resource settings, where limited access to effective screening and prevention contributes to elevated rates of morbidity and mortality [2]. In response, international guidelines now recommend high-risk human papillomavirus (hrHPV) DNA testing as the primary method for cervical cancer screening due to its high sensitivity [[3], [4], [5]]. However, the high prevalence of transient HPV infections poses a significant challenge for clinical management, necessitating accurate triage strategies to identify women at true risk of high-grade cervical lesions and avoid unnecessary procedures.

Although liquid-based cytology (e.g., ThinPrep cytology test, TCT) is widely used for triaging HPV-positive women, its performance is highly variable, with reported sensitivity ranging from 55% to 94% and specificity from 63.8% to 75.5% [6]. This variability underscores the urgent need for more reliable molecular biomarkers. Several emerging options are under investigation, including E6/E7 mRNA testing [7], p16/Ki-67 dual staining (DS) [8], extended HPV genotyping [9], and host/viral gene methylation panels [10,11]. The latest WHO guidelines encourage the incorporation of such novel technologies to improve risk stratification and optimize clinical management. Nevertheless, there remains no consensus on the optimal triage strategy for HPV-positive women to minimize unnecessary colposcopy referrals and overtreatment.

Persistent infection with hrHPV is a necessary cause of cervical carcinogenesis, with HPV integration into the host genome serving as a frequent and critical molecular event associated with malignant transformation. Studies have shown that the integration of HPV-16 and HPV-18 DNA into the host genome occurs at an early stage of CC progression [12]. While most HPV infections are transient and cleared by the immune system, integrated HPV DNA disrupts host genomic stability, promotes oncogene expression and drives progression from cervical intraepithelial neoplasia (CIN) to invasive cancer [13]. Emerging evidence highlights that HPV integration may serve as a biomarker for high-grade lesions and poor clinical outcomes [[14], [15], [16]]. HPV16 integration contributes to cervical oncogenesis through interrupting tumor suppressor genes and inducing chromosome instability [17]. Despite its biological significance, the clinical utility of HPV integration testing as a routine triage tool remains underexplored.

Given the limitations of current diagnostic methods and the urgent need for more reliable early detection strategies for CC, this study aimed to evaluate the clinical validity of HPV integration detection as a risk-stratification biomarker within a screening cohort of HPV-positive women. We assessed its prevalence, its association with high-grade lesions, and its performance both alone and in combination with HPV genotyping. We further propose a novel integrated triage strategy aiming to improve the precision of colposcopy referrals, maximize early detection of CIN3+, and minimize invasive procedures in low-risk individuals. Our findings support the use of HPV integration status as a key tool in the era of precision screening, potentially informing future guideline updates and clinical practice.

## 2 Methods

### 2.1 Study design

**Fig. 1:**
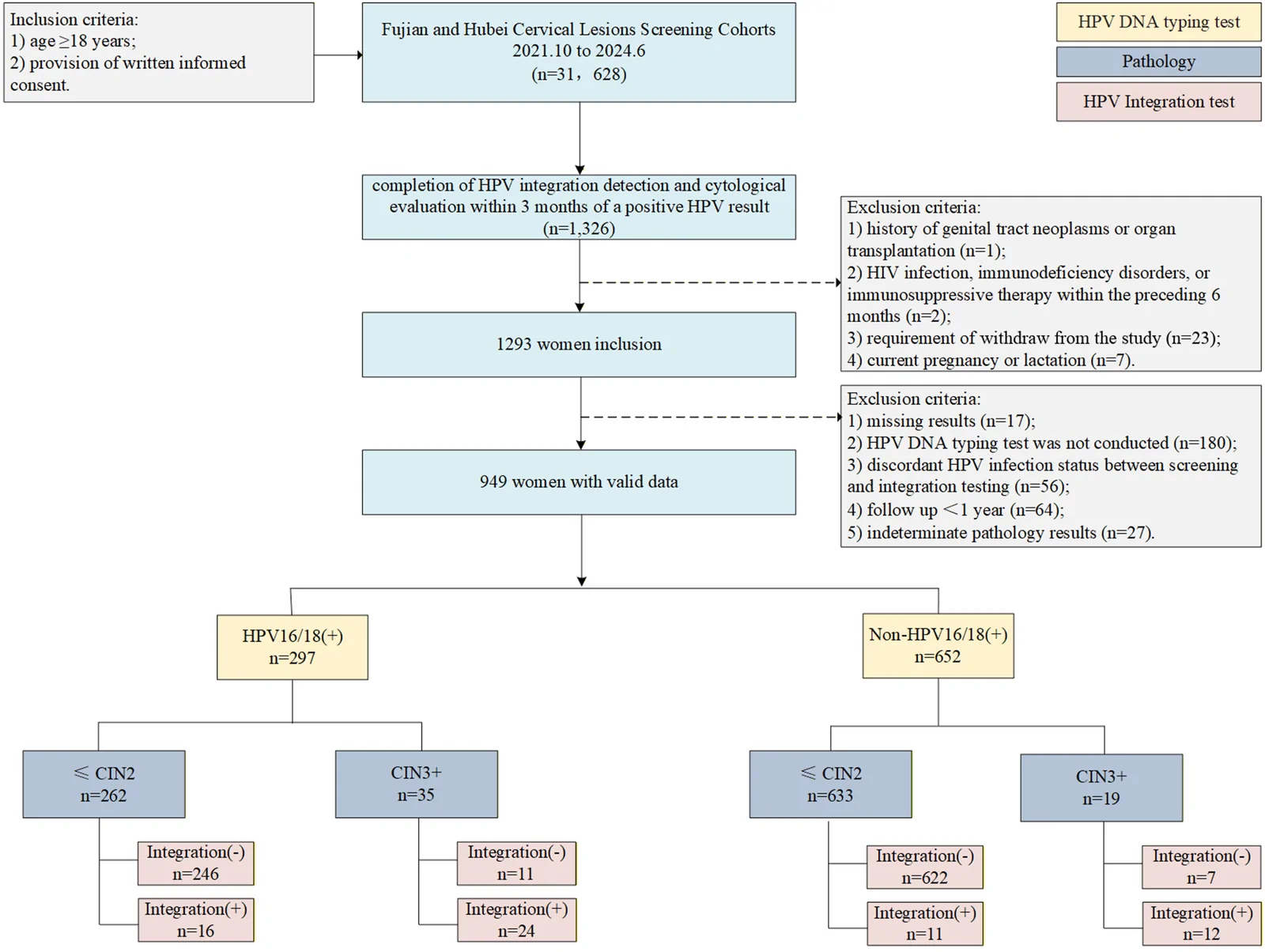
Study flowchart. Abbreviations: HPV, human papillomavirus; CIN3+, cervical intraepithelial neoplasia grade 3 or worse; ≤CIN2 group comprised histologically confirmed benign or inflammatory findings, CIN1, and CIN2.

From the Fujian-Hubei Cervical Lesion Screening Cohort (N = 31,628), we enrolled 1326 women undergoing simultaneous cervical cancer screening and HPV integration testing (October 2021–June 2024). After applying eligibility criteria (Fig. 1), 949 participants who underwent colposcopy-guided cervical biopsy and had valid HPV integration results and definitive histopathological diagnoses comprised the final analytic cohort. Patients diagnosed with CIN 3 or worse (CIN 3+, including CIN3, adenocarcinoma in situ, or invasive carcinoma) received management per American Society for Colposcopy and Cervical Pathology (ASCCP) consensus guidelines [4]. Ethical approval was obtained from the Institutional Review Board of Fujian Maternity and Child Health Hospital (Approval number: 2024KLR09009). All participants provided signed informed consent.

### 2.2 HPV DNA detection

The HPV Genotyping Kit for 23 Types (PCR-Reverse Dot Blot, Yaneng® Biosciences) was used to identify 23 genotypes in exfoliated cervical cells, including 13 high-risk (HR) HPV genotypes (HPV16, -18, −31, −33, −35, −39, −45, −51, −52, −56, −58, −59, and −68) and 5 possible high-risk HPV genotypes (HPV53, -66, −73, −82, and −83), and 5 low-risk HPV genotypes (HPV6, -11, −42, −43, and −81). All the detection procedures strictly followed the manufacturer's instructions. Analyses stratified infections by HPV16/18 positivity status and HPV pattern (single vs. multiple infections).

### 2.3 HPV integration detection

Following the methodology outlined in previous research, we employed high-throughput viral integration detection (HIVID) to conduct tests for HPV integration [15,18]. Briefly, genomic DNA was extracted from exfoliated cervical cells, and libraries were constructed using a multiplex probe-based capture system targeting the entire genomes of HPV types. Sequencing was performed on an Illumina platform. To ensure assay reproducibility, a spike-in reference control (standardized fragments) was added to each sample, requiring a recovery rate of ≥0.01% for a valid test. The mean sequencing quality reached a Q30 of ≥90.0%. Integration status was determined automatically by a dedicated, pre-defined bioinformatics pipeline to eliminate observer bias. A sample was categorized as integration-positive if ≥ 6 unique virus-host chimeric reads (after removing PCR duplicates) were identified at a specific genomic site [14,19]. This assay provides genotype-specific results, enabling precise identification of the integrated HPV type even in cases of multiple infections. Samples exhibiting discrepancies between the HPV integration test and PCR-RDB genotyping results were excluded from the analysis.

### 2.4 Cytological diagnoses

Cytological results were categorized per the Bethesda System: negative for intraepithelial lesion or malignancy (NILM), ASC-US, low-grade squamous intraepithelial lesion (LSIL), atypical glandular cells (AGC), atypical squamous cells—cannot exclude HSIL (ASC-H), and high-grade squamous intraepithelial lesion (HSIL).

### 2.5 Histological diagnoses

Histopathological findings from cervical biopsies served as the reference standard for disease status. Histological findings were classified as benign or inflammatory findings, CIN1, CIN2, or CIN3+. CIN3+ was defined as CIN3, adenocarcinoma in situ, or invasive cervical carcinoma. All biopsies were independently reviewed by two blinded pathologists, with discrepancies resolved by a third expert. Participants without a definitive histopathological diagnosis were excluded from the analysis.

### 2.6 Statistical analysis

Continuous variables are presented as medians (interquartile range [IQR]) and categorical variables as counts (%). Group comparisons utilized Kruskal-Wallis rank-sum tests for continuous data and Pearson's χ^2^/Fisher's exact tests for categorical variables. Diagnostic performance metrics (sensitivity, specificity, positive/negative predictive values [PPV/NPV]) were calculated as percentages. McNemar's test compared diagnostic concordance between HPV integration and cytology. Associations with CIN3+ were examined using maximum-likelihood logistic regression. Odds ratios (ORs) were obtained from univariable models. Adjusted odds ratios (aORs) were obtained from separate models adjusted for age group. ORs are reported with 95% profile-likelihood confidence intervals (CIs), and two-sided P values were calculated using likelihood-ratio tests. Bootstrap and Bayesian logistic regression were used as sensitivity analyses. Bootstrap 95% bias-corrected and accelerated (BCa) CIs were calculated from 10,000 participant-level resamples. Bayesian models used the same covariates and reference categories as the primary models, with weakly informative normal priors for the regression coefficients and intercept. Four independent chains were run, each with 4000 burn-in and 4000 retained iterations. Results are reported as posterior median ORs with 95% credible intervals (CrIs). Convergence was assessed using R-hat and effective sample size. Statistical significance in the primary analysis was defined as a two-sided P < 0.05. All analyses were performed using R version 4.5.2.

## 3 Results

### 3.1 Demographic and clinical characteristics by HPV integration status

The baseline characteristics of the 949 HPV-positive women are summarized in Table 1. HPV integration positivity was identified in 6.6% (63/949) of participants. No significant age difference was observed between HPV integration-negative (median: 38 years, IQR: 31-50) and HPV integration-positive groups (median: 37 years, IQR: 31-47; p = 0.327; Table 1). Notably, HPV16/18 genotypes were significantly more prevalent in HPV integration-positive women compared to their HPV integration-negative counterparts (63.5% vs. 29.0%, p < 0.001; Table 1). HPV integration status correlated strongly with the severity of cytological lesions. Among HPV-positive women, HPV integration positivity rates increased significantly across cytological categories: 5.1% (30/592) in the NILM group, 7.2% (22/306) in the ASC-US/AGC/LSIL group, and 21.6% (11/51) in the ASC-H/HSIL group (p < 0.001). Pathologically, HPV integration-positive women exhibited markedly higher proportions of high-grade lesions (CIN3+: 57.1% vs. 2.0%; p < 0.001; Table 1), confirming a positive association between HPV integration status and cervical lesion severity.

**Table 1:** Patient characteristics according to HPV integration status.

Characteristics	Parameters	HPV integration negative, N = 886	HPV integration positive, N = 63	p value
Age	Median (IQR)	38 (31, 50)	37 (31, 47)	0.327
HPV genotyping	HPV16/18+	257 (29.0%)	40 (63.5%)	<0.001
	Non-HPV16/18+	629 (71.0%)	23 (36.5%)	
HPV pattern	Single infection	623 (70.3%)	49 (77.8%)	0.208
	Multiple infection	263 (29.7%)	14 (22.2%)	
Cytology	NILM	562 (63.4%)	30 (47.6%)	<0.001
	ASC-US/AGC/LSIL	284 (32.1%)	22 (34.9%)	
	ASC-H/HSIL	40 (4.5%)	11 (17.5%)	
Pathology	≤CIN2	868 (98.0%)	27 (42.9%)	<0.001
	CIN3+	18 (2.0%)	36 (57.1%)	

Abbreviations:NILM, negative for intraepithelial lesion or malignancy; ASC-US, atypical squamous cells of undetermined significance; LSIL, low-grade squamous intraepithelial lesion; ASC-H, atypical squamous cells cannot exclude high-grade squamous intraepithelial lesion; HSIL, high-grade squamous intraepithelial lesion; AGC, atypical glandular cells; CIN, cervical intraepithelial neoplasia.

Note: ≤CIN2 comprised histologically confirmed benign or inflammatory findings (n = 461), CIN1 (n = 267), and CIN2 (n = 167). CIN3+ includes CIN3, adenocarcinoma in situ, and invasive carcinoma.

The distribution of HPV integration by genotype is presented in Supplementary Table S1. HPV integration was detected in 28.3% (15/53) of HPV18-positive samples, 11.5% (25/218) of HPV16-positive samples, 4.4% (9/205) of HPV52-positive samples, and 1.4% (2/145) of HPV58-positive samples. Findings for less common genotypes are provided descriptively in Supplementary Table S1 because of the small group sizes.

### 3.2 Risk factors for CIN3+

After adjusting for age, the multivariable logistic regression showed that HPV integration positivity had the strongest association with CIN3+ (aOR = 68.1, 95% CI: 34.4–140.9; p < 0.001; Table 2). Non-HPV16/18 infections were associated with lower odds of CIN3+ than HPV16/18 infections (aOR = 0.2; 95% CI: 0.1–0.4; p < 0.001; Table 2). Further analysis showed that patients with HPV16/18 infection combined with HPV integration-positive had the highest risk (aOR = 33.1, 95% CI: 14.1–83.4; p < 0.001; Table 2). Non-HPV16/18 infections with HPV integration-positive also had higher odds of CIN3+ than the reference group (aOR = 31.8; 95% CI: 11.1–98.0; p < 0.001; Table 2). Multiple HPV infections showed no significant association with CIN3+ risk compared to single infections (aOR = 1.0, 95% CI: 0.5–1.8; p = 0.982; Table 2). Additionally, HPV infection patterns were found to have varying impacts on CIN3+ risk when combined with HPV integration status. Notably, the combination of single infection and HPV integration-positive presented a substantially elevated risk (aOR = 121.8; 95% CI: 51.1–323.0; p < 0.001; Table 2).

**Table 2:** Logistic regression analysis of factors associated with CIN3+.

Characteristics		Univariable logistic regression		Age-adjusted logistic regression	
		OR (95% CI)	p value	aOR (95% CI)	Adjusted p value
Age	≤45	Ref			
	>45	0.5 (0.2 - 0.9)	0.019		
HPV integration	HPV integration-	Ref		Ref	
	HPV integration+	64.3 (33.0 - 130.2)	<0.001	68.1 (34.4 - 140.9)	<0.001
HPV genotyping	HPV16/18+	Ref		Ref	
	Non-HPV16/18+	0.2 (0.1 - 0.4)	<0.001	0.2 (0.1 - 0.4)	<0.001
HPV pattern	Single infection	Ref		Ref	
	Multiple infection	1.0 (0.6 - 1.8)	0.942	1.0 (0.5 - 1.8)	0.982
combination of HPV genotyping & HPV integration	HPV16/18+ and HPV integration-	Ref		Ref	
	Non-HPV16/18+ and HPV integration-	0.3 (0.1 - 0.7)	0.004	0.3 (0.1 - 0.7)	0.006
	HPV16/18+ and HPV integration+	33.6 (14.4 - 83.6)	<0.001	33.1 (14.1 - 83.4)	<0.001
	Non-HPV16/18+ and HPV integration+	24.4 (9.0 - 69.6)	<0.001	31.8 (11.1- 98.0)	<0.001
combination of HPV pattern & HPV integration	Single infection and HPV integration-	Ref		Ref	
	Single infection and HPV integration+	121.4 (51.4 - 318.4)	<0.001	121.8 (51.1 - 323.0)	<0.001
	Multiple infection and HPV integration-	3.0 (1.2 - 8.0)	0.021	2.9 (1.1 - 7.7)	0.026
	Multiple infection and HPV integration+	57.7 (16.0 - 209.7)	<0.001	66.4 (17.9 - 251.7)	<0.001

**Abbreviations:** CI, confidence interval; CIN3+, cervical intraepithelial neoplasia grade 3 or worse; HPV, human papillomavirus; OR, odds ratio; aOR, adjusted odds ratio.

**Note:** ORs were estimated using maximum-likelihood logistic regression. The 95% CIs were calculated using the profile-likelihood method, and two-sided P values were obtained using likelihood-ratio tests. Except for the age-group analysis, adjusted models included age group (≤45 vs > 45 years) as a covariate. The reference category is indicated as “Reference”.

To assess the robustness of the results, we performed bootstrap resampling with 10,000 iterations and Bayesian analysis. The two sensitivity analyses produced similar estimates for HPV integration positivity (bootstrap aOR = 68.1, 95% BCa CI: 31.7–138.7; Bayesian posterior median OR = 64.3, 95% CrI: 32.7–130.4; Supplementary Table S3), supporting its strong association with CIN3+.

### 3.3 Stratified analysis by cytologic diagnosis

Stratification by cytology revealed pronounced disparities in risk of CIN3+ (Table 3). Among the 17 women with NILM cytology and histologically confirmed CIN3+, 13 (76.5%) were HPV integration-positive and 4 (23.5%) were HPV integration-negative. Among the 22 women with ASC-US/AGC/LSIL cytology and histologically confirmed CIN3+, 12 (54.5%) were HPV integration-positive and 10 (45.5%) were HPV integration-negative. Among the 15 women with ASC-H/HSIL cytology and histologically confirmed CIN3+, 11 (73.3%) were HPV integration-positive and 4 (26.7%) were HPV integration-negative.

**Table 3:** Association of CIN 3+ with HPV integration status stratified by cytologic diagnosis.

Cytologic Diagnosis	≤CIN2	CIN3+	p value
**NILM, n(%)**			
HPV integration-	558 (97.0%)	4 (23.5%)	<0.001
HPV integration+	17 (3.0%)	13 (76.5%)	
HPV16/18+ and HPV integration-	169 (94.4%)	2 (15.4%)	<0.001
HPV16/18+ and HPV integration+	10 (5.6%)	11 (84.6%)	
Non-HPV16/18+ and HPV integration-	389 (98.2%)	2 (50.0%)	0.003
Non-HPV16/18+ and HPV integration+	7 (1.8%)	2 (50.0%)	
HPV16+ and HPV integration-	118 (96.7%)	1 (16.7%)	<0.001
HPV16+ and HPV integration+	4 (3.3%)	5 (83.3%)	
HPV18+ and HPV integration-	51 (89.5%)	1 (14.3%)	<0.001
HPV18+ and HPV integration+	6 (10.5%)	6 (85.7%)	
Single infection and HPV integration-	405 (97.1%)	2 (14.3%)	<0.001
Single infection and HPV integration+	12 (2.9%)	12 (85.7%)	
Multiple infection and HPV integration-	153 (96.8%)	2 (66.7%)	0.108
Multiple infection and HPV integration+	5 (3.2%)	1 (33.3%)	
**ASC-US/AGC/LSIL, n(%)**			
HPV integration-	274 (96.5%)	10 (45.5%)	<0.001
HPV integration+	10 (3.5%)	12 (54.5%)	
HPV16/18+ and HPV integration-	68 (91.9%)	6 (42.9%)	<0.001
HPV16/18+ and HPV integration+	6 (8.1%)	8 (57.1%)	
Non-HPV16/18+ and HPV integration-	206 (98.1%)	4 (50.0%)	<0.001
Non-HPV16/18+ and HPV integration+	4 (1.9%)	4 (50.0%)	
HPV16+ and HPV integration-	50 (89.3%)	6 (50.0%)	0.005
HPV16+ and HPV integration+	6 (10.7%)	6 (50.0%)	
HPV18+ and HPV integration-	18 (100.0%)	0 (0.0%)	<0.001
HPV18+ and HPV integration+	0 (0.0%)	3 (100.0%)	
Single infection and HPV integration-	182 (96.3%)	4 (30.8%)	<0.001
Single infection and HPV integration+	7 (3.7%)	9 (69.2%)	
Multiple infection and HPV integration-	92 (96.8%)	6 (66.7%)	0.008
Multiple infection and HPV integration+	3 (3.2%)	3 (33.3%)	
**ASC-H/HSIL, n(%)**			
HPV integration-	36 (100.0%)	4 (26.7%)	<0.001
HPV integration+	0 (0.0%)	11 (73.3%)	
HPV16/18+ and HPV integration-	9 (100.0%)	3 (37.5%)	0.009
HPV16/18+ and HPV integration+	0 (0.0%)	5 (62.5%)	
Non-HPV16/18+ and HPV integration-	27 (100.0%)	1 (14.3%)	<0.001
Non-HPV16/18+ and HPV integration+	0 (0.0%)	6 (85.7%)	
HPV16+ and HPV integration-	6 (100.0%)	3 (37.5%)	0.031
HPV16+ and HPV integration+	0 (0.0%)	5 (62.5%)	
HPV18+ and HPV integration-	3 (100%)	0 (0.0%)	
HPV18+ and HPV integration+	0 (0.0%)	0 (0.0%)	-
Single infection and HPV integration-	28 (100.0%)	2 (18.2%)	<0.001
Single infection and HPV integration+	0 (0.0%)	9 (81.8%)	
Multiple infection and HPV integration-	8 (100.0%)	2 (50.0%)	0.091
Multiple infection and HPV integration+	0 (0.0%)	2 (50.0%)	
Total**, n(%)**			
HPV16/18+ and HPV integration-	246 (93.9%)	11 (31.4%)	<0.001
HPV16/18+ and HPV integration+	16 (6.1%)	24 (68.6%)	
Non-HPV16/18+ and HPV integration-	622 (98.3%)	7 (36.8%)	<0.001
Non-HPV16/18+ and HPV integration+	11 (1.7%)	12 (63.2%)	
HPV16+ and HPV integration-	174 (94.6%)	10 (38.5%)	<0.001
HPV16+ and HPV integration+	10 (5.4%)	16 (61.5%)	
HPV18+ and HPV integration-	72 (92.3%)	1 (10.0%)	<0.001
HPV18+ and HPV integration+	6 (7.7%)	9 (90.0%)	
Single infection and HPV integration-	615 (97.0%)	8 (21.1%)	<0.001
Single infection and HPV integration+	19 (3.0%)	30 (78.9%)	
Multiple infection and HPV integration-	253 (96.9%)	10 (62.5%)	<0.001
Multiple infection and HPV integration+	8 (3.1%)	6 (37.5%)	

Abbreviations:NILM, negative for intraepithelial lesion or malignancy; ASC-US, atypical squamous cells of undetermined significance; LSIL, low-grade squamous intraepithelial lesion; ASC-H, atypical squamous cells cannot exclude high-grade squamous intraepithelial lesion; HSIL, high-grade squamous intraepithelial lesion; AGC, atypical glandular cells; CIN, cervical intraepithelial neoplasia.

Note: CIN3+, cervical intraepithelial neoplasia grade 3 or worse.

### 3.4 Diagnostic performance of HPV integration detection

HPV integration detection demonstrated superior specificity (97.0% vs. 64.3%, p < 0.001) and positive predictive value (PPV: 57.1% vs. 10.4%, p < 0.001) compared to cytology for CIN3+ detection (Table 4). Critically, HPV integration detection exhibited significantly higher accuracy than cytology (95.3% vs. 64.5%, p < 0.001). The sensitivity between the two methods was comparable (66.7% versus 68.5%, p = 0.847). Subgroup analyses, which were stratified by HPV genotypes and age, corroborated these findings: among women positive for HPV16/18, HPV integration detection achieved a PPV of 60.0% compared to 21.0% for cytology (p < 0.001); in women over the age of 45, the PPV was 38.9% compared to 6.6% (p = 0.007).

**Table 4:** Diagnostic performance of HPV integration detection compared to cytology for CIN3+ detection.

		Positivity, No./Total patients	Detection of CIN3+					
			Positivity, % (95% CI)	Accuracy, % (95% CI)	Sensitivity, % (95% CI)	Specificity, % (95% CI)	PPV, % (95% CI)	NPV, % (95% CI)
HPV + women (n = 949)	**HPV Integration**	63/949	6.6 (5.2 - 8.5)	95.3 (93.7 - 96.4)	66.7 (53.4 - 77.8)	97.0 (95.7 - 98.0)	57.1 (48.3 - 65.5)	97.97 (95.63 - 99.21)
	TCT	357/949	37.6 (34.5 - 40.8)	64.5 (61.4 - 67.5)	68.5 (55.3 - 79.3)	64.3 (61.1 - 67.3)	10.4 (6.7 - 15.3)	97.13 (94.51 - 98.65)
	p value		<0.001	<0.001	0.847	<0.001	<0.001	0.253
HPV16/18+ women (n = 297)	**HPV Integration**	40/297	13.5 (9.9 - 18.0)	90.9 (87.1 - 93.7)	68.6 (52.0 - 81.5)	93.9 (90.3 - 96.2)	60.0 (44.6 - 73.7)	95.72 (92.50 - 97.59)
	TCT	105/297	35.4 (30.0 - 41.1)	67.7 (62.2 - 72.7)	62.9 (46.3 - 76.8)	68.3 (62.5 - 73.7)	21.0 (14.3 - 29.7)	93.23 (88.76 - 96.00)
	p value		<0.001	<0.001	0.656	<0.001	<0.001	0.212
Non-HPV16/18+ women (n = 652)	**HPV Integration**	23/652	3.5 (2.3 - 5.3)	97.2 (95.7 - 98.3)	63.2 (41.0 - 80.9)	98.3 (96.9 - 99.0)	52.2 (33.0 - 70.8)	98.89 (97.72 - 99.46)
	TCT	252/652	38.7 (34.9 - 42.5)	63.0 (59.3 - 66.7)	79.0 (56.7 - 91.5)	62.6 (58.7 - 66.2)	6.0 (3.6 - 9.6)	99.00 (97.46 - 99.61)
	p value		<0.001	<0.001	0.257	<0.001	<0.001	0.826
HPV + women (age ≤45 years old) (n = 642)	**HPV Integration**	45/642	7.0 (5.2 - 9.3)	95.2 (93.2 - 96.6)	65.9 (51.1 - 78.1)	97.3 (95.7 - 98.4)	64.4 (49.8 - 76.8)	97.49 (95.90 - 98.47)
	TCT	266/642	41.4 (37.6 - 45.4)	61.4 (57.6 - 65.1)	70.5 (55.8 - 81.9)	60.7 (56.7 - 64.5)	11.7 (8.3 - 16.1)	96.54 (94.18 - 97.97)
	p value		<0.001	<0.001	0.670	<0.001	<0.001	0.350
HPV + women (age >45 years old) (n = 307)	**HPV Integration**	18/307	5.9 (3.6 - 9.3)	95.4 (92.5 - 97.3)	70.0 (39.7 - 89.2)	96.3 (93.5 - 97.9)	38.9 (20.3 - 61.4)	98.96 (96.99 - 99.65)
	TCT	91/307	29.6 (24.7 - 35.2)	71.0 (65.7 - 75.8)	60.0 (31.3 - 83.2)	71.4 (66.0 - 76.2)	6.6 (3.6 - 13.7)	98.15 (95.34 - 99.28)
	p value		<0.001	<0.001	0.655	<0.001	0.007	0.384

Abbreviations:PPV, positive predictive value; NPV, negative predictive value; CIN3+, cervical intraepithelial neoplasia grade 3 or worse.

### 3.5 Comparative diagnostic performance of combined strategies for CIN3+ detection

As illustrated in Table 5, the combined diagnostic strategies demonstrated distinct performance characteristics for CIN3+ detection. The integration of cytology with HPV16/18 genotyping resulted in a sensitivity of 92.6% (95% CI: 82.5–97.1) and a specificity of 44.3% (95% CI: 41.0–47.5), alongside an accuracy of 47.0% (95% CI: 43.8–50.2) and a PPV of 9.1% (95% CI: 7.0–11.8). Conversely, the combination of HPV integration detection with HPV16/18 genotyping significantly enhanced specificity to 69.5% (95% CI: 66.4–72.4; p < 0.001) and accuracy to 70.5% (95% CI: 67.5–73.3; p < 0.001), while maintaining a comparable sensitivity of 87.0% (95% CI: 75.6–93.6; p = 0.526) and achieving a higher PPV of 14.7% (95% CI: 11.2–19.0; p = 0.014). Similarly, the combination of HPV integration detection with cytology preserved high sensitivity (92.6%, 95% CI: 82.5–97.1; p = 1.0) and achieved moderate accuracy (64.0%, 95% CI: 61.0–67.1; p < 0.001), albeit with lower specificity compared to the combination of HPV integration with HPV16/18 genotyping (62.4%, 95% CI: 59.1–65.5; p < 0.001). Importantly, all strategies maintained high NPV, with no statistically significant differences in NPV observed among the approaches (p = 0.725). These results indicate that the incorporation of HPV integration detection enhances diagnostic performance.

**Table 5:** Combined diagnostic performance of HPV integration detection with cytology or HPV16/18 genotyping for CIN3+ detection.

	Positivity		Accuracy		Sensitivity		Specificity		PPV		NPV	
	% (95% CI)	p value	% (95% CI)	p value	% (95% CI)	p value	% (95% CI)	p value	% (95% CI)	p value	% (95% CI)	p value
Cytology and HPV16/18 genotyping	57.9 (54.7- 61.0)	Ref	47.0 (43.8 - 50.2)	Ref	92.6 (82.5 - 97.1)	Ref	44.3 (41.0 - 47.5)	Ref	9.1 (7.0 - 11.8)	Ref	99.00 (97.46 - 99.61)	Ref
HPV integration and HPV16/18 genotyping	33.7 (30.8 - 36.8)	<0.001	70.5 (67.5 - 73.3)	<0.001	87.0 (75.6 - 93.6)	0.526	69.5 (66.4 - 72.4)	<0.001	14.7 (11.2 - 19.0)	0.014	98.89 (97.72 - 99.46)	1
HPV integration and Cytology	40.8 (37.7 - 43.9)	<0.001	64.0 (61.0 - 67.1)	<0.001	92.6 (82.5 - 97.1)	1	62.4 (59.1 - 65.5)	<0.001	12.9 (10.0 - 16.6)	0.068	99.29 (98.18 - 99.72)	0.725

Abbreviations:PPV, positive predictive value; NPV, negative predictive value; CIN3+, cervical intraepithelial neoplasia grade 3 or worse.

### 3.6 Risk stratification of CIN3+ by different screening strategies

**Fig. 2:**
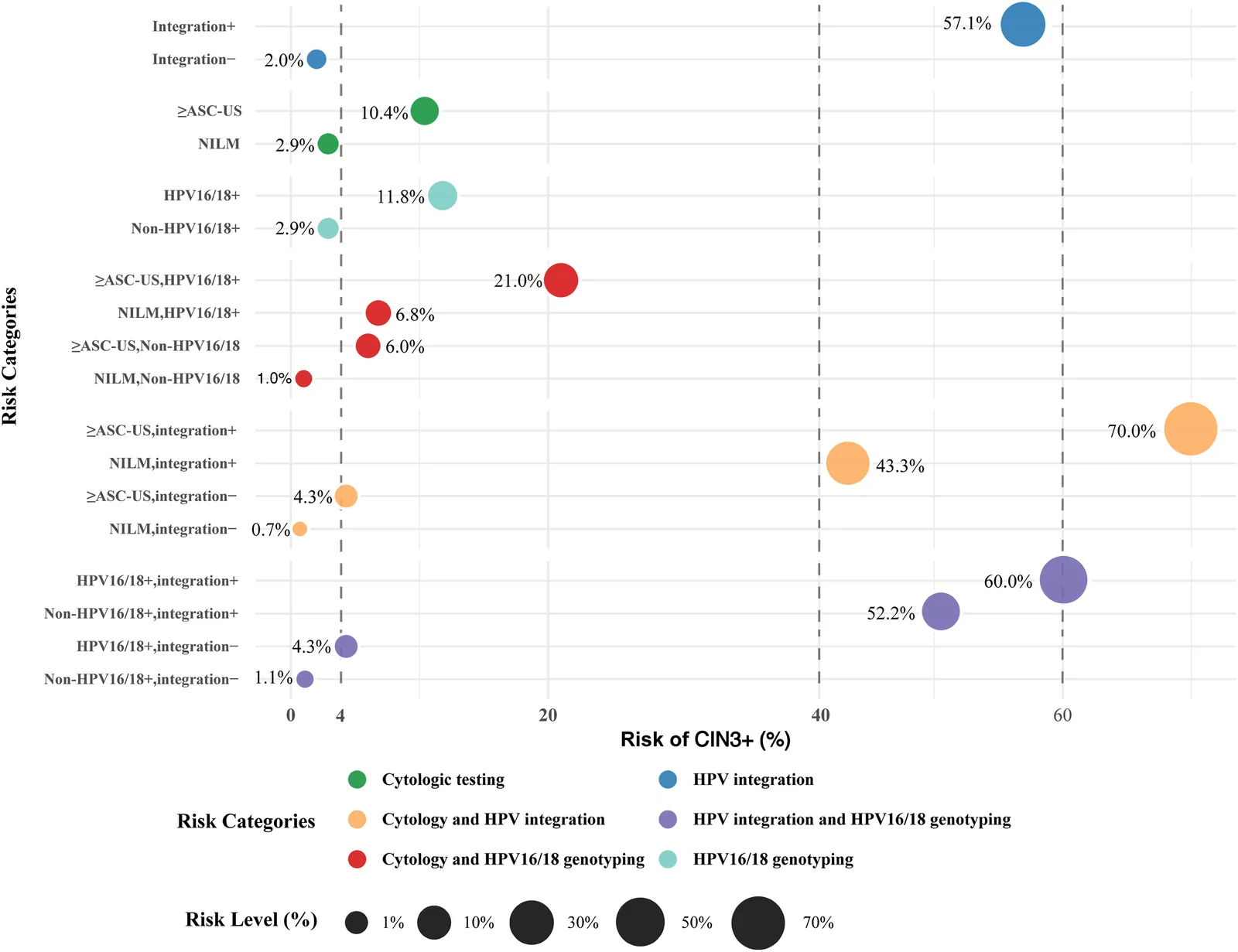
The risk of CIN3+ for women stratified by different screening strategies. Abbreviations: HPV, human papillomavirus; ≥ASC-US (ASC-US or worse) includes ASC-US, LSIL, ASC-H, HSIL, and AGC; CIN3+, cervical intraepithelial neoplasia grade 3 or worse; NILM, negative for intraepithelial lesion or malignancy.

As depicted in Fig. 2, distinct screening strategies demonstrated significant variations in identifying the risk of CIN3+. Among HPV-positive participants, those identified as HPV integration-positive exhibited the highest direct risk of CIN3+ at 57.1%, markedly surpassing the 10.4% risk observed in the ≥ASC-US group. It is noteworthy that HPV integration-negative individuals comprised a significantly larger proportion of the cohort compared to the NILM group (93.4% [886/949] vs. 62.4% [592/949]), yet both groups showed comparable low direct risks of CIN3+ (2.0% vs. 2.9%, respectively). In cases that were HPV16/18+ and HPV integration-negative, the risk escalated to 4.3%, whereas those that were HPV16/18+ and integration-positive had a 60.0% risk. When compared to the ASCCP-defined low-risk population (NILM and HPV16/18-negative, 1.0% [4/400]), the NILM and integration-negative subgroup displayed a similarly low CIN3+ risk (0.7% [4/562]) but encompassed a larger population size (59.2% vs. 42.1%, Table S2). Among all combinations analyzed, the ≥ASC-US and integration-positive subgroup presented the highest risk of CIN3+ at 70.0%.

## 4 Discussion

HPV integration into the host genome has been observed in cervical carcinogenesis and is associated with greater lesion severity [20]. Our findings elucidate the critical role of HPV integration detection in refining risk stratification and clinical management for HPV-positive women. Analysis of The Cancer Genome Atlas (TCGA) data revealed >80% HPV integration prevalence in HPV-associated CC [21], while a prospective cohort study reports HPV integration rate of 10.7% in HR HPV-positive women [15]. In HPV 16-positive precancerous cervical lesions, HPV integration rate was 7.4% [22]. Our cohort demonstrated a slightly lower HPV integration prevalence (6.6%, 63/949), potentially attributable to population-specific characteristics. HPV16/18-positive individuals exhibited significantly higher HPV integration propensity. Notably, HPV18 demonstrated disproportionately high HPV integration rate (28.3%) relative to its prevalence. Consistent with regional epidemiological patterns [23], HPV52/58—despite their high prevalence in Fujian Province—showed low integration frequencies (4.4% and 1.4%, respectively). No HPV integration events were detected in HPV45, -66, −68, −73, or −82 positive cases. Different types of HR-HPVs differ in the extent to which they trigger chromosomal instability in host cells, in the frequency of their integration in advanced lesions, and the time it takes for CIN 3 lesions to progress to invasive cancer [24]. Thus, further studies are needed to clarify.

Our analysis revealed that 77.8% of HPV integration-positive cases occurred in single infections, with multiple HPV infections accounting for only 22.2%. The effect of multiple infections on CIN3+ risk did not differ from that of single infections. However, there is no general consensus on whether multiple HPV infections increase the incidence of HPV consolidation or increase the risk of CC. Although some prospective studies suggest synergistic carcinogenic effects of multiple HR-HPV infections [25], several studies have published reports comparable cervical cancer risks between single and multiple infections [26,27]. Mingxia Li et al. demonstrated that differential risks between single and multiple infections depend critically on specific genotypes and co-infection patterns [28]. Our results showed lower CIN3+ risk in multiple infections with HPV integration-positive compared to single infections with HPV integration-positive, suggesting potential interactions between viral competition, host immune responses, and synergistic oncoprotein activities. These findings underscore the need for mechanistic studies on poly viral carcinogenesis while maintaining clinical vigilance in managing multiple HR-HPV infections.

Multivariate analysis confirmed HPV integration as a robust independent factor of CIN3+ risk, with bootstrap resampling and Bayesian validation reinforcing the association's stability while mitigating overfitting concerns. A previous study by our team found HPV integration to be an independent risk factor for residual/recurrent CIN in postoperative HPV-positive women [29]. In this study, we found HPV integration status was strongly associated with advanced lesions even in cytologically normal or low-grade cases. This aligns with established evidence of increasing HPV integration frequencies paralleling lesion severity [30,31], solidifying its biological relevance in cervical carcinogenesis. Collectively, these findings position HPV integration status as a critical biomarker for identifying high-risk individuals with underlying CIN3+ lesions.

In the context of global cervical screening paradigms, HPV-based primary screening has gained prominence since 2019. As the world's largest developing country, early screening remains the most important intervention to prevent CC in our country. Although HPV primary screening is highly sensitive, its screening positive population is much higher than cytology screening, and cytology is still needed for triage. However, China faces unique challenges in implementing HPV primary screening due to limited cytology resources and variable quality control [32], compounded by cytology's inherent false-positive limitations [33]. Our comparative analysis revealed superior performance of HPV integration testing over cytology in specificity (96.98% vs. 64.25%) and positive predictive value (57.14% vs. 10.36%), while maintaining comparable sensitivity (66.67% vs. 68.52%). Age and HPV genotyping are key factors influencing HPV clearance and lesion recurrence. Previous studies have shown a significant correlation between HPV infection and age. The HPV integration-based strategy demonstrated consistent diagnostic accuracy across age groups and HPV16/18 infection status. The combined HPV16/18 genotyping plus HPV integration strategy achieved enhanced specificity (69.50%) and PPV (14.69%) compared to cytology-based approaches, extending ASCCP's genotype-specific risk assessment paradigm through molecular integration status. Critically, the low-risk NILM/integration-negative subgroup (0.7% CIN3+ risk) encompassed 59.2% of the cohort, suggesting potential for safely reducing surveillance intensity in majority populations. Our data support a sequential "HPV Type + Integration" strategy as an objective molecular triage to further differentiate risk within HPV16/18-positive or cytologically normal populations, potentially prioritizing colposcopy for those with high risk while avoiding over-intervention in integration-negative cases. These findings corroborate prior evidence supporting molecular triage superiority over cytology, while revealing a crucial limitation of morphological assessment: even low-grade cytology (NILM/ASC-US) cases with positive integration carried substantial CIN3+ risk (57.14%), challenging the assumption of cytological normality as low-risk. HPV integration detection promises to be an objective, sensitive, and practical triage strategy for CC screening.

Our findings support the use of HPV integration testing to create a more precise risk-based triage algorithm. Rather than merely reducing the number of referrals, the goal is to increase the accuracy of referrals – that is, to increase the proportion of women referred who actually have high-grade disease. The HPV integration-based strategy achieved this by effectively reclassifying a large low-risk population (NILM/integration-negative, 59.2% of cohort) with a very low risk of CIN3+ (0.7%), who could be managed with surveillance, thereby allowing clinical resources to be focused on the smaller subset of integration-positive women who carry the vast majority of disease risk. Furthermore, we emphasize that cases showing high-risk HPV integration (particularly HPV18) alongside negative histology should be managed as a "high-risk state" rather than a mere false positive. These discordant cases warrant intensified surveillance or further diagnostic evaluation to prevent the potential underdiagnosis of occult lesions.

### 4.1 Strengths and limitations

This study possesses several notable strengths. First, rigorous multivariable adjustment for key confounders strengthens the validity of observed associations. Second, the diagnostic performance of HPV integration testing was systematically validated through both bootstrap resampling and Bayesian analytical frameworks, ensuring robustness against overfitting. Finally, the inclusion of subgroup analyses across age strata and HPV16/18 infection status demonstrates consistent test performance, reinforcing its clinical utility in diverse patient profiles. However, there are several limitations to consider. First, the cross-sectional design does not permit assessment of the temporal relationship between HPV integration and cervical lesion development. Second, the relatively low proportion of HPV integration-positive cases (6.6%) and the absence of longitudinal data preclude causal inferences about the temporal role of integration in disease progression. This limits the statistical precision and generalizability of subgroup analyses to different populations. Specifically, the low regional prevalence of certain genotypes, such as HPV45, resulted in a limited sample size for these subsets. Although no integration events were detected among the six cases of HPV45 infection, this "null effect" is insufficient to draw definitive conclusions or provide a stable statistical estimate regarding the integration characteristics of this genotype. Third, the current cost of HPV integration testing remains higher than that of routine HPV genotyping and may limit wider implementation. Although its higher specificity may reduce downstream colposcopy and biopsy use, the present study did not include a formal health-economic analysis. Its cost-effectiveness therefore requires further evaluation. In order to find optimal treatment strategies, larger longitudinal studies are needed in the future to validate these findings and establish standards. Future large-scale longitudinal studies are imperative to validate clinical utility and establish standardized protocols.

## 5 Conclusion

In conclusion, our findings underscore HPV integration as a significant independent risk factor for CIN3+. When combined with genotyping, it enables highly precise risk stratification within the HPV-positive population. These findings support HPV integration status as an adjunctive molecular marker for triage among HPV-positive women, particularly for identifying women with a higher concurrent probability of CIN3+. Its cost-effectiveness and impact on downstream clinical management warrant further evaluation.

**Table:** Abbreviations

ASC-US	atypical squamous cells of undetermined significance
CC	cervical cancer
CIN	cervical intraepithelial neoplasia
HPV	human papillomavirus
HSIL	high-grade squamous intraepithelial lesion
LSIL	low-grade squamous intraepithelial lesion
NILM	negative for intraepithelial lesion or malignant neoplasm
NPV	negative predictive value
PPV	positive predictive value

## CRediT authorship contribution statement

**Xite Lin:** Writing – original draft, Data curation. **Yue Wang:** Writing – review & editing, Visualization, Validation, Data curation. **Nan Yu:** Methodology, Formal analysis, Data curation. **Dingjie Wang:** Writing – review & editing, Data curation. **Liang Wang:** Supervision, Software. **Xiaoyuan Huang:** Writing – review & editing, Investigation, Formal analysis, Conceptualization. **Binhua Dong:** Resources, Project administration, Methodology, Funding acquisition, Conceptualization. **Pengming Sun:** Writing – review & editing, Resources, Funding acquisition.

## Ethics declaration

Written informed consent to take part in the study and to publish the article has been obtained from all participants or their legal representatives. The privacy rights of participants have been observed.

This study was performed in compliance with relevant laws, regulatory frameworks and guidelines where the research took place. This study was approved by the Institutional Review Board of Fujian Maternity and Child Health Hospital. (Approval No. 2024KLR09009)

## Availability of data and materials

The data that support the findings of our study are in an anonymized form available from the corresponding author upon reasonable request and following the data protection regulations.

## Ethics approval and consent to participate

Ethics Committee of Fujian Maternity and Child Health Hospital (No. 2024KLR09009). All participants provided written informed consent.

## Consent for publication

Not applicable.

## Funding

This work was supported by the following grants. National Key R&D Program of China (grant no. 2025YFC2708402). 10.13039/501100001809National Natural Science Foundation of China (grant no. 82271658). Major Scientific Research Program for Young and Middle-aged Health Professionals of Fujian Province, China (grant no. 2021ZQNZD011). Fujian Province Central Government-Guided Local Science and Technology Development Project (grant no. 2023L3019). 10.13039/501100003392Fujian Provincial Natural Science Foundation of China (grant no. 2025J01199, no. 2022J011040). Fujian Provincial Health Commission's Mid-career & Young Core Talent Training Program (grant no. 2024GGA061); Startup Fund for scientific research, 10.13039/501100013795Fujian Medical University (grant no. 2024QH2048).

## Declaration of competing interest

The authors declare no competing financial interests or personal relationships that could have influenced the work reported in this manuscript. This study was supported by public research grants from the National Key R&D Program of China (grant no. 2025YFC2708402), National Natural Science Foundation of China (grant no. 82271658), and Fujian Province government funds (grant no. 2021ZQNZD011; 2023L3019; 2025J01199, 2022J011040; 2024GGA061). Wuhan Kindvace Biotechnology Co., Ltd. provided standardized sequencing services on a fee-for-service basis. No commercial entities were involved in study design, data analysis, or manuscript preparation.

## Data Availability

Data will be made available on request.
