# Supplementary material for: HPV integration as a triage tool for HPV-positive women in cervical cancer screening
Source: Tumour Virus Res. 2026 Sep 1;22:200351. doi: 10.1016/j.tvr.2026.200351 (PMC13571999; doi:10.1016/j.tvr.2026.200351)
Supplement: Supplementary file 1 [file mmc1.docx]

| **Table S1.** Prevalence and Distribution of HPV Integration by Genotype. | | | |
| --- | --- | --- | --- |
| **HPV Infection Type** | **Total(No.)** | **HPV integration** | **Percentage(%）** |
| HPV16 | 218 | 25 | 11.5 |
| HPV18 | 53 | 15 | 28.3 |
| HPV31 | 6 | 1 | 16.7 |
| HPV33 | 18 | 2 | 11.1 |
| HPV35 | 12 | 1 | 8.3 |
| HPV39 | 27 | 2 | 7.4 |
| HPV45 | 6 | 0 | 0.0 |
| HPV51 | 30 | 1 | 3.3 |
| HPV52 | 205 | 9 | 4.4 |
| HPV56 | 44 | 1 | 2.3 |
| HPV58 | 145 | 2 | 1.4 |
| HPV59 | 39 | 3 | 7.7 |
| HPV68 | 48 | 0 | 0.0 |
| HPV53 | 54 | 1 | 1.9 |
| HPV66 | 49 | 0 | 0.0 |
| HPV73 | 9 | 0 | 0.0 |
| HPV82 | 12 | 0 | 0.0 |

| **Table S2.** Risk Stratification of CIN3+ by Cytology, HPV Integration, and HPV16/18 Status. | | | | |
| --- | --- | --- | --- | --- |
| **Characteristic** |  | **Women, No. (%)** | **CIN3+, No.** | **Risk of CIN3+, %** |
| HPV integration | HPV integration- | 886 (93.4) | 18 | 2.0 |
|  | HPV integration+ | 63 (6.6) | 36 | 57.1 |
| Cytologic testing | NILM | 592 (62.4) | 17 | 2.9 |
|  | ≥ASC-US | 357 (37.6) | 37 | 10.4 |
| HPV16/18 genotyping | Non-HPV16/18+ | 652 (68.7) | 19 | 2.9 |
|  | HPV16/18+ | 297 (31.3) | 35 | 11.8 |
| Cytology and HPV16/18 genotyping | NILM and Non-HPV16/18+ | 400 (42.1) | 4 | 1.0 |
|  | NILM and HPV16/18+ | 192 (20.2) | 13 | 6.8 |
|  | ≥ASC-US and Non-HPV16/18+ | 252 (26.6) | 15 | 6.0 |
|  | ≥ASC-US and HPV16/18+ | 105 (11.1) | 22 | 21.0 |
| Cytology and HPV integration | NILM and HPV integration- | 562 (59.2) | 4 | 0.7 |
|  | NILM and HPV integration+ | 30 (3.2) | 13 | 43.3 |
|  | ≥ASC-US and HPV integration- | 324 (34.1) | 14 | 4.3 |
|  | ≥ASC-US and HPV integration+ | 33 (3.5) | 23 | 70.0 |
| HPV integration and HPV16/18 genotyping | Non-HPV16/18+ and HPV integration- | 629 (66.3) | 7 | 1.1 |
|  | Non-HPV16/18+ and HPV integration+ | 23 (2.4) | 12 | 52.2 |
|  | HPV16/18+ and HPV integration- | 257 (27.1) | 11 | 4.3 |
|  | HPV16/18+ and HPV integration+ | 40 (4.2) | 24 | 60.0 |

Abbreviations: NILM, negative for intraepithelial lesion or malignancy; ASC-US, atypical squamous cells of undetermined significance; LSIL, low-grade squamous intraepithelial lesion; ASC-H, atypical squamous cells cannot exclude high-grade squamous intraepithelial lesion; HSIL, high-grade squamous intraepithelial lesion; AGC, atypical glandular cells.

Note: ≥ASC-US (ASC-US or worse) includes ASC-US, LSIL, ASC-H, HSIL, and AGC.

| **Table S3.** Bootstrap and Bayesian Sensitivity Analyses of Factors Associated With CIN3+. | | | |
| --- | --- | --- | --- |
| **Characteristics** | | **Bootstrap OR (95% BCa CI)** | **Bayesian posterior OR (95% CrI)** |
| Age | ≤45 | Ref | Ref |
|  | >45 | 0.5 (0.2–0.9) | 0.5 (0.2–0.9) |
| HPV integration | HPV integration- | Ref | Ref |
|  | HPV integration+ | 68.1 (31.7–138.7) | 64.3 (32.7–130.4) |
| HPV genotyping | HPV16/18+ | Ref | Ref |
|  | Non-HPV16/18+ | 0.2 (0.1–0.4) | 0.2 (0.1–0.4) |
| HPV pattern | Single infection | Ref | Ref |
|  | Multiple infection | 1.0 (0.5–1.8) | 1.0 (0.5–1.8) |
| combination of HPV genotyping & HPV integration | HPV16/18+ and HPV integration- | Ref | Ref |
|  | Non-HPV16/18+ and HPV integration- | 0.3 (0.1–0.8) | 0.2 (0.1–0.6) |
|  | HPV16/18+ and HPV integration+ | 33.1 (12.8–84.4) | 29.9 (12.9–72.7) |
|  | Non-HPV16/18+ and HPV integration+ | 31.8 (9.4–98.6) | 27.4 (9.8–80.0) |
| combination of HPV pattern & HPV integration | Single infection and HPV integration- | Ref | Ref |
|  | Single infection and HPV integration+ | 121.8 (45.8–327.2) | 100.4 (43.6–245.5) |
|  | Multiple infection and HPV integration- | 2.9 (1.02–8.5) | 2.4 (0.97–5.9) |
|  | Multiple infection and HPV integration+ | 66.4 (14.3–244.3) | 46.3 (12.8–161.7) |

**Abbreviations:** BCa, bias-corrected and accelerated; CI, confidence interval; CrI, credible interval; OR, odds ratio.

**Note:** Bootstrap results are presented as ORs from the original-sample maximum-likelihood estimates with 95% BCa CIs based on 10,000 participant-level resamples. Bayesian results are presented as posterior median ORs with 95% CrIs. Except for the age-group analysis, all models were adjusted for age group (≤45 vs >45 years). Bootstrap P values were not calculated because the bootstrap analysis was used for interval estimation. Values are reported to one decimal place, except for lower interval limits close to 1.0, which are reported to two decimal places.
